# Comparison of strength profile representations using musculoskeletal models and their applications in robotics

**DOI:** 10.3389/frobt.2023.1265635

**Published:** 2024-01-09

**Authors:** Sheila Sutjipto, Marc G. Carmichael, Gavin Paul

**Affiliations:** UTS Robotics Institute, University of Technology Sydney, Sydney, NSW, Australia

**Keywords:** modeling and simulating humans, human strength estimation, human factors, physical human-robot interaction, musculoskeletal model

## Abstract

Musculoskeletal models provide an approach towards simulating the ability of the human body in a variety of human-robot applications. A promising use for musculoskeletal models is to model the physical capabilities of the human body, for example, estimating the strength at the hand. Several methods of modelling and representing human strength with musculoskeletal models have been used in ergonomic analysis, human-robot interaction and robotic assistance. However, it is currently unclear which methods best suit modelling and representing limb strength. This paper compares existing methods for calculating and representing the strength of the upper limb using musculoskeletal models. It then details the differences and relative advantages of the existing methods, enabling the discussion on the appropriateness of each method for particular applications.

## 1 Introduction

There has been recent interest in the use of models to interpret the effect of musculoskeletal variability on human physical performance. Such models have primarily been applied in human factors and ergonomics to design tasks that prevent musculoskeletal disorders and improve productivity by considering human capability. Leveraging information gained from musculoskeletal models, the frontiers of human-robot interaction integrate the knowledge of human capability to alleviate physical burden during manual handling (Peternel et al., 2019); in healthcare, for the assessment and estimation of human capability (Carmichael and Liu, 2015); and more recently, to provide assistance via robotic solutions (Carmichael and Liu, 2011; Lai et al., 2019; Petrič et al., 2019).

Human capability can be defined using various quantities. However, physical strength is an appropriate measure when considering the utilization of robotics to alleviate the physical burden during laborious tasks. Anthropometric surveys of human strength often systematically measure the strength of isolated joints in the limb, such as the shoulder (Riemann et al., 2010; Baillargeon et al., 2022), elbow (Hernandez et al., 2015), wrist (Decostre et al., 2015; Chimera et al., 2021), and the hips and knee (Kordi et al., 2017). Although relatively simple to perform, these strength measurements only consider a single joint in the limb, usually limited to movement within anatomical planes and constrained under isometric or isokinetic conditions.

Instead of strength at the joint, *whole limb* strength measured at the end-point of the limb (i.e., hand or foot) is a more relevant measure of physical capability for real-world interactions. Whole limb strength may be estimated by considering the isolated joint strength capacities of a limb, and similar methods are used in robotics to calculate the end-point loading capacity of a robot arm. This makes sense in robotics since robot joint strength is typically uncoupled.

Such strategies for human strength estimation predominately rely on empirical measurements of Maximum Voluntary Contractions (MVC), or model-based approximations, to estimate whole-limb strength (Sasaki et al., 2010; Hernandez et al., 2015; Rezzoug et al., 2021). The main drawback of this approach is the resources and time necessary to acquire good quality data as it requires consideration of factors such as managing body posture and sensor placement and handling fatigue. Additionally, applying this joint-strength approach when calculating whole-limb strength does not take into consideration the complex inter-joint and inter-muscular factors that affect the strength of the limb due to multi-articular muscles.

**FIGURE 1:**
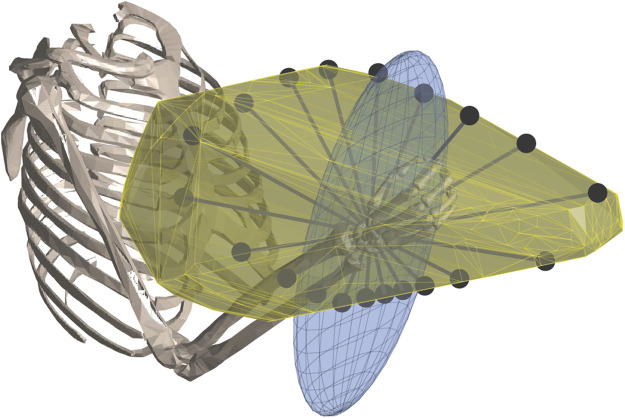
Examples of strength profiles computed and represented by rays (black dots), a polytope (yellow), and an ellipsoid (blue).

The whole-limb strength can be better estimated using a musculoskeletal model that characterises the kinematic, dynamic and musculo-tendon properties of a limb. The advantage of utilizing musculoskeletal models is that, given a suitably accurate model, the limb strength can be estimated in arbitrary limb configurations, and kinematic and dynamic conditions. Additionally, musculoskeletal models also consider multi-articular muscles, agonist-antagonist co-contraction, and other biomechanical factors that affect whole-limb strength. Several methods have been developed to utilize musculoskeletal models for estimating the strength at the end of a human limb, with different approaches to calculating and representing this strength being proposed (Carmichael and Liu, 2013; Hernandez et al., 2018; Petrič et al., 2019; Sohn et al., 2019; Skuric et al., 2022). Figure 1 shows three methods commonly used for strength representation at the hand.

Despite the variety of existing methods for calculating and representing whole-limb strength using musculoskeletal models, the suitability of these methods in different applications requires further investigation.

In this work, an existing musculoskeletal model is employed to facilitate the comparison of commonly used methods for calculating and representing the whole-limb strength of a limb. This paper is not intended to be a comprehensive review of musculoskeletal modelling, or the models for strength estimation. We instead directly compare three different methods for computing and representing strength, focusing on their differences, advantages, and limitations. Section 2 provides a necessary overview of musculoskeletal models. Section 3 details existing methods of using musculoskeletal models to calculate whole-limb strength. In Section 4, the strength models are applied to demonstrate their differences. Finally, in Section 5, trade-offs in the methods and the suitability of different strength estimation methods for various robotics use cases is discussed.

## 2 Musculoskeletal modelling

Musculoskeletal models use modelling techniques and computational tools to represent and simulate complex neuromuscular systems. They provide a means of studying these systems to gain insights into their inner workings without direct measurement. This section provides a non-exhaustive summary of common musculoskeletal modelling methods that form the basis of the strength estimation methods discussed in Section 3.

### 2.1 Limb kinematics and dynamics

The human skeletal system can be modelled as a system of rigid bodies representing the body segments and the joints connecting them. The kinematics and dynamics of such a system can be ascertained using methods similar to those utilized in robotics.

The configuration of the limb is described by a vector of generalised joint coordinates, $q={\left[q_{1},q_{2},\ldots ,q_{k}\right]}^{T}\in R^{k}$ . The pose of the limb end-point, **x** is computed using forward kinematics (1).

$$x=f\left(q\right)$$ (1)

The spatial velocity of the end-point, $\dot{x}$ is calculated using the partial derivative of the forward kinematic relationship (2). The Jacobian matrix, $J\left(q\right)\in R^{n\times k}$ relates joint-space generalised coordinate velocities to Cartesian linear and angular velocities.

$$\dot{x}=\left(\frac{\partial f}{\partial q}\right)\dot{q}=J\left(q\right)\dot{q}$$ (2)

**FIGURE 2:**
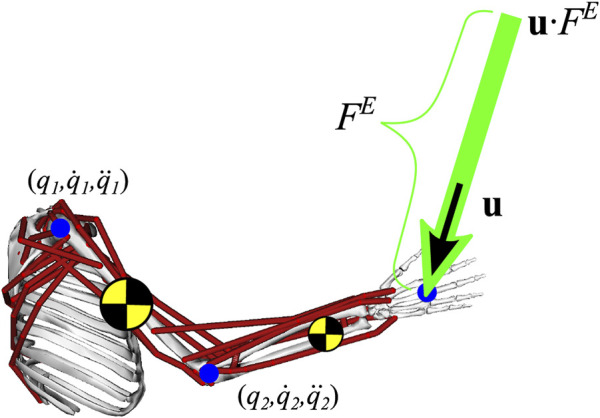
Strength representations are generated using a musculoskeletal model representing a human. An optimization model calculates the maximum magnitude of external force (*S*_*P*_ = max [*F*_*E*_]) the human can oppose (Carmichael and Liu, 2013).

Each rigid link in the model possesses attributes such as mass and inertial properties. Incorporating these parameters, the dynamic equation of the system can be expressed as (3) where **H** is the inertia matrix, **C** are centrifugal and Coriolis effects and ***τ***_*G*_ are joint torques due to gravity. An external force applied to the limb end-point is included, represented as the product of a unit vector representing the force direction, **u**, and the scalar magnitude of the external force, *F*_*E*_, as shown in Figure 2.

$$H\left(q\right)\ddot{q}+C\left(q,\dot{q}\right)\dot{q}+\tau_{G}\left(q\right)=\tau_{M}+J{\left(q\right)}^{T}u\cdot F_{E}$$ (3)

where the vector, ***τ***_*M*_, represents the joint torques resulting from muscle forces.

### 2.2 Musculo-tendon units

Actuation of the human body is achieved by the contraction of muscles that are attached to the skeleton via tendons. Musculoskeletal models commonly represent this muscular actuation by musculo-tendon units (MTUs), with each unit representing their respective muscle (or group of muscles) and the tendons connecting them to the skeleton. The MTU model allows the force output of the muscle to be computed as a function of its length, contraction velocity, muscle fibre pennation angle, and other state variables and physiological parameters. Various MTU models have been developed (Zajac, 1989; Schutte et al., 1993; Thelen et al., 2003) with most models derived from the Hill muscle model (Hill, 1938).

The force output of a muscle is a combination of the active and passive force output exhibited by the muscle. The amount of active force output a muscle produces is represented by its activation, *a* ∈ [0, 1]. An activation of *a* = 0 represents a completely passive muscle, while *a* = 1 represents the muscle at full contraction. It is noted that muscles cannot generate or relax their force output instantaneously. The activation dynamics of a muscle can be modelled with a first-order differential equation relating the rate of change in activation to the muscles’ excitation, often utilizing different time constants for activation and deactivation. However, activation dynamics are often not considered when conducting strength analyses. Prior work has shown that static and dynamic optimization can produce similar results (Anderson and Pandy, 2001) and that the system is generally assumed to be static (McKay et al., 2007; Sohn et al., 2019; Aldini et al., 2021).

During peak isometric force, tendons exhibit minimal stretch, around 3%, as shown by Zajac (1989). Thus, with the assumption that the tendon is rigid, the length of the muscle fibre can be geometrically related to the total MTU length. By defining this length and contraction speed as *l* and $\dot{l}$ , respectively, and assuming the tendon is rigid, the MTU tensile force can be expressed as (4) comprising a passive force component and an active force component dependent on the activation, *a*.

The force-length and force-velocity relationships for these elements have been modelled as normalized curves that are functions of the normalized muscle fibre length and normalized muscle fibre contraction velocity. These non-linear relationships are captured by functions ${\tilde{f}}_{m}^{P}\left(l,\dot{l}\right)$ and ${\tilde{f}}_{m}^{A}\left(l,\dot{l}\right)$ , corresponding to normalized passive and active force output respectively (De Sapio et al., 2005). The scalar value, *F* ^0^ represents the maximum peak isometric force of the muscle, *m*, often estimated from the physiological cross-sectional area of the muscle.

$$f_{m}=\underset{passivef_{m}^{P}}{\underbrace{{\tilde{f}}_{m}^{P}\left(l,\dot{l}\right)F_{m}^{0}}}+\underset{activef_{m}^{A}}{\underbrace{{\tilde{f}}_{m}^{A}\left(l,\dot{l},a_{m}\right)F_{m}^{0}a_{m}}}$$ (4)

The joint torque, ***τ***_*M*_ generated from the muscle forces is calculated as.

$$\tau_{M}=\tau_{P}+\tau_{A}\text{, where}$$ (5)

$$\tau_{P}=-L^{T}f_{P}$$ (6)

$$\tau_{A}=-L^{T}K_{A}a$$ (7)

where $f_{P}={\left[f_{1}^{P},f_{2}^{P},\ldots ,f_{m}^{P}\right]}^{T}$ represents the vector of passive muscle forces, **f**_*A*_ = **K**_*A*_ **a** represents the vector of active muscle forces, $a={\left[a_{1},a_{2},\ldots ,a_{m}\right]}^{T}$ represents the vector of muscle activations, $L\left(q\right)\in R^{m\times k}$ is the Jacobian matrix containing partial derivatives that relate the rate of change in MTU lengths to the rate of change in joint coordinates, i.e., $\dot{l}=L\dot{q}$ where $l={\left[l_{1},l_{2},\ldots ,l_{m}\right]}^{T}\in R^{m}$ . Using energy principles, **L** ^*T*^ maps the muscle forces to joint torques. The negative sign in Equations 6, 7 is due to the convention of the partial derivative in **L** relating a positive change in joint coordinate to a positive change in MTU length. However, the MTU activation causes muscles to shorten, not lengthen. The right-hand side of the active component shown in (4) can be represented as a diagonal matrix, **K**_*A*_, containing the MTU forces generated per unit muscle activation (De Sapio et al., 2005).

## 3 Methods of computing whole limb strength

We define the problem of computing whole limb strength as the following: given the state of the limb (or body), compute the force-producing capabilities at the end-point of the limb.

Considering the human limb represented as a musculoskeletal model (MM) like that shown in Figure 2, this problem can be formulated as: given the state of the musculoskeletal model, (**q**, $\dot{q}$ , $\ddot{q}$ ), determine the force-producing capabilities at the end-point of the limb (e.g., *F* ^*E*^), subject to system constraints such as the system’s equation of motion (3).

To compare methods for computing whole limb strength, it is important first to acknowledge that there are multiple ways to represent this strength. In this work, strength representations are classified into three types: *Point*, *Polytope*, and *Ellipsoid*. The following subsections will detail existing methods for computing strength from the musculoskeletal model for each representation.

### 3.1 Points

The simplest method for representing strength is to consider a single direction of interest and to compute the maximal force-producing capabilities of the end-point of the limb in that chosen direction. Early works iterated on this approach at the limb end-point to obtain strength in discrete directions corresponding to push and pull, up and down, and left and right (Badler et al., 1993).

With each of the aforementioned axes being orthonormal, there are too few axes to ascertain a more accurate strength representation of the hand. In Cartesian space, this would result in 6 points that describe the strength in the positive and negative *x*, *y* and *z*-axes. However, choosing vectors along a plane formed by these axes allows a 2D profile to be constructed. Discretizing the space around the hand leads to a desire to calculate strength along a vector of interest. Determining the location where the ray intersects with the boundary of the strength profile, can be referred to as *ray-shooting*. This boundary is dictated by the maximal force-producing capabilities of the limb end-point in all Cartesian directions.

One application of the ray-shooting method to compute strength at the end-point of the limb is presented in Carmichael and Liu (2013). Starting with the equation of motion of the musculoskeletal model (3), and substituting (5), (6), (7), the equation of motion of the limb can be represented as:

$$J^{T}u\cdot F_{E}=\tau_{B}+L^{T}K_{A}a$$ (8)

where elements that are independent of muscle activation or the external force ( $H\ddot{q}$ , **C**, ***τ***_*G*_ and ***τ***_*P*_) are combined into a single joint torque vector, ***τ***_*B*_ (9).

$$\tau_{B}=H\ddot{q}+C+\tau_{G}-\tau_{P}$$ (9)

With the musculoskeletal model equation of motion in the form above, computing the strength can be formulated as an optimisation problem to find the maximum scalar value of *F* ^*E*^.

Once **u** is defined, the problem of computing maximum strength becomes an optimisation problem to maximise the scalar, *F*_*E*_. These methods search the space of feasible muscle activations to find solutions that maximise *F*_*E*_ subject to the system’s equation of motion (8) being satisfied.

The underlying approach developed in Carmichael and Liu (2013) leverages (8) and implements a linear optimisation stated as.

$$Objective:\;\max\left[F_{E}\right]$$ (10)

$$\text{Subject to}:\;J^{T}u\cdot F_{E}=\tau_{B}+L^{T}K_{A}\cdot a$$ (11)

0≤a≤1 (12)

$$F_{E}<F_{\text{max}}$$ (13)

where muscle activations are bound between 0 ≤ **a** ≤ 1 and *F*_max_ is set to 300N (Carmichael and Liu, 2013). In Carmichael and Liu (2013), this optimisation was solved using linear programming techniques.

Similar methods exist that take an alternative approach, for example, simplifying the computation by ignoring elements contributing to the bias torques, ***τ***_*B*_ (Valero-Cuevas, 2009; Sohn et al., 2019).

### 3.2 Polytopes

When a strength profile is required (instead of strength in discrete directions), a strength *polytope* can be generated. A polytope (polygon in 2D, polyhedra in 3D) is a visually intuitive representation of strength across different directions.

A polytope can be created by repeatedly utilising ray-shooting methods in directions spanning Cartesian space and then connecting the data points to produce the polytope. For example, a 2D strength profile can be visualized by computing the strength about the hand in discretized directions existing in frontal, coronal and sagittal planes (Carmichael and Liu, 2011; Hernandez et al., 2015). The accuracy of the polytope to represent the *true* strength profile of the limb depends on the number of strength calculations made. Therefore, this approach to polytope generation, although simple, can be computationally demanding if a high-fidelity profile is required.

These polytopes are defined by *feasible sets* in the muscle, torque or Cartesian spaces where the muscle activations have undergone a linear transformation that is only valid for a particular state of the musculoskeletal model. For representing strength capability, the output space of interest is the Cartesian space where the set of feasible wrenches exists. Early works include generating such polytopes by directly measuring forces in Cartesian space (Valero-Cuevas et al., 1998) or mapping maximum joint torque measurements into the Cartesian space (Sasaki et al., 2011).

Alternatively, other methods exist that compute convex polytopes using techniques developed for closed-chains (Bouchard et al., 2010; Gouttefarde and Krut, 2010), which similarly determines the available wrench set considering minimum and maximum values in the input space. The hyper-plane shifting method (HPSM) exploits the nature of convex polytopes of which the force set is (Skuric et al., 2022). This method uses hyperplanes to find the intersection of half-spaces to define the polytope. Depending on the use case, it is useful to obtain the vertices of the polytope, which can be achieved with existing methods (Bremner et al., 1998).

Although an exact solution provides the most accurate result, the complexity of achieving such a task for a high dimensional real-time system is intractably time-consuming, thus limiting its use to offline applications (Skuric et al., 2022). Subsequently, various approximation algorithms have been implemented to approximate the set of feasible wrenches of the limb. Ultimately, these approximation algorithms generate the vertices of the polytope whilst employing a particular method of computing strength as described in Section 3.

Rather than performing a more exhaustive search of the solution space, the iterative convex hull algorithm presented in Skuric et al. (2022) approximates the polytope by strategically generating vectors to maximize muscle force. The approach presented grows the polytope by performing the optimization to find the maximum strength at the desired vector, then, based upon a threshold, determines whether a new polytope vertex is found or whether the point is close enough to the existing face to be considered to lie on that face. This can reduce the time required to generate the polytope since it is based on a required level of accuracy.

### 3.3 Ellipsoidal representations

An alternative representation of whole limb strength is the ellipsoid. Ellipsoidal representations are a popular graphical tool used for analyzing kinematic chains (e.g., robot manipulators) through measures such as manipulability (Yoshikawa, 1984) and dynamic manipulability (Yoshikawa, 1985).

When examining the capability of a robotic manipulator, these aforementioned measures are based on the manipulator Jacobian, **J**, as derived in Eq. 1. For manipulability, if *rank* **J** < *m*, the manipulator is considered to be in a singular state and cannot satisfy any arbitrary $\dot{x}$ , thus implying a system low manipulability. A scalar value can be associated with this manipulability descriptor with the following equation,

$$w=\sqrt{det\left({JJ}^{T}\right)}$$ (14)

This value corresponds to the volume of an ellipsoid represented in Cartesian space, which has been mapped from a unit sphere in the joint space of the manipulator, thus satisfying $\left\|a\right\|=1$ .

$${\dot{q}}^{T}\dot{q}\leq 1$$ (15)

$${\left(J^{-1}\dot{x}\right)}^{T}\left(J^{-1}\dot{x}\right)\leq 1$$ (16)

$${\dot{x}}^{T}{\left(JJ^{T}\right)}^{-1}\dot{x}\leq 1$$ (17)

These ellipsoidal concepts, commonly applied in robotics, can be applied to musculoskeletal models to evaluate the strength of the limb. Conceptually, this class of representation considers a unit sphere in the *m*-dimensional muscle activation space and how this transforms into an ellipsoid of force-production in the limb Cartesian space, which is defined as the Muscular Force Manipulability Ellipsoid (MFME) (Petrič et al., 2019).

Using (8), and assuming a unit sphere in the muscle activation space (i.e., $\left\|a\right\|=1$ , following similarly to Eq. 17, and no bias torques (***τ***_*B*_ = 0), since its effect only translates the centre of the ellipsoid), an expression can be derived for the corresponding ellipsoid (18).

$$\begin{array}{l} a^{T}a\leq 1\\ {\left({\left(L^{T}K_{A}\right)}^{-1}J^{T}uF_{E}\right)}^{T}\left({\left(L^{T}K_{A}\right)}^{-1}J^{T}uF_{E}\right)\leq 1\\ F_{E}u^{T}{\left(\left(J^{-T}L^{T}K_{A}\right){\left(J^{-T}L^{T}K_{A}\right)}^{T}\right)}^{-1}uF_{E}\leq 1 \end{array}$$ (18)

Using singular value decomposition, the inner component of (18), $\left(J^{-T}L^{T}K_{A}\right){\left(J^{-T}L^{T}K_{A}\right)}^{T}$ , can be decomposed to obtain the properties necessary to define an ellipsoid. From this process, the eigenvectors and eigenvalues of the matrix can be obtained; these are the orthonormal vectors corresponding to the principal axes of the ellipsoid, and the corresponding magnitudes of these principal axes are defined by the singular values.

In contrast to polytope representations, it is evident that there are necessary assumptions embedded into the ellipsoid formulation that violate physiological constraints. Namely, the resulting ellipsoid has been mapped from a unit sphere in the muscle activation space. These limitations are detailed in the discussion.

## 4 Comparison of methods and representations

This section compares three different techniques: the ray-shooting method presented in Carmichael and Liu (2013), the strength polytope derived from Gouttefarde and Krut (2010) and implemented in Skuric et al. (2022), and the strength ellipsoid described in Petrič et al. (2019).

A musculoskeletal model comprising 50 muscles and 7 degrees of freedom (DoF) Saul et al. (2015); McFarland et al. (2019) was used to compare these approaches to generate strength and illustrate their respective representations. For simplification, the model has been reduced to 4DoF encompassing motions at the shoulder (elv_angle, shoulder_elv, shoulder_rot) and elbow (elbow_flexion). The computer model was modified from Saul et al. (2015) as described by McFarland et al. (2019) to include an updated range of motion at the shoulder, and ligaments models representing the glenohumeral and coracohumeral ligaments. Furthermore, the muscle model was updated with force-length and tendon curves matching the original model’s respective curves (Millard et al., 2013). This model was utilised with the OpenSim (Seth et al., 2018) application programming interface via MATLAB (Mathworks, Inc., Natick, MA, United States of America). This contains and facilitates the calculation of physiological variables, such as **K**_*A*_, **L**, and **J**, required for the estimating the force at the end-point.

The limb dynamics are set to reflect the state of the human upper limb during MVC, i.e., $\ddot{q}=\dot{q}=0$ . The torque due to gravity possesses the effect of translating the centre of the representation for both polytopes or ellipsoids, consequently it is omitted as it does not alter the profile of the representation. Additionally, it is well understood that muscle fatigue affects the output capability of the human. Within literature, several models have been proposed to predict fatigue (Xia and Frey Law, 2008; Ma et al., 2009; Peternel et al., 2016), and such approaches have been employed in human-robot collaboration frameworks (Lorenzini et al., 2019; Peternel et al., 2019). In this work the effects of fatigue on limb strength are ignored and assume that the limb is capable of performing peak isometric strength, since the intent is to highlight the differences between the methods and representations rather than the effect of fatigue. Unless otherwise specified, the polytopes generated using the ray-shooting method utilized the results obtained from using HPSM to choose the ray directions for easier comparison. In other words, the vertices from the HPSM polytope are normalized to form the unit vectors used in the ray-shooting method.

The presented results are based on conditions observed during MVC and rely on commonly employed assumptions in musculoskeletal models. This condition is utilised since MVC is frequently employed when assessing the force generation capacity of a muscle or muscle group, and can subsequently be used to normalize EMG signals for obtaining a muscle activation. As mentioned in 2.2, this assumes that the arm is static, i.e., $\ddot{q}=\dot{q}=0$ , the tendons are rigid (Zajac, 1989), the torque due to gravity is ignored since it is not a function of the muscle activation, and activation dynamics are omitted (Anderson and Pandy, 2001). By removing these factors, this work solely focuses on discerning the differences between the methods employed during the process of estimating end-point capability.

### 4.1 Effect of enforcing a unit circle input space on strength capacity

**FIGURE 3:**
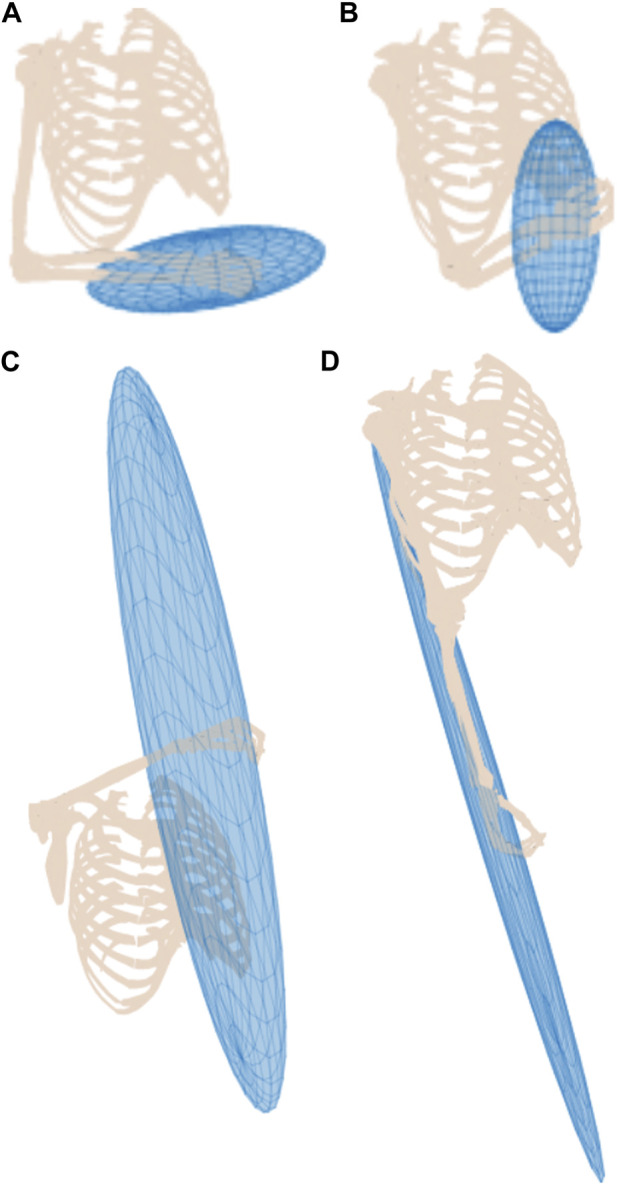
Musculoskeletal model in four different poses with their respective muscular manipulability ellipsoids. Higher ellipsoid isotropy is shown in **(A, B)**, compared to the isotropy in **(C, D)**.

A series of arm configurations with their corresponding MFME is illustrated in Figure 3. This figure demonstrates how the ellipsoid changes based on the configuration of the arm. In configurations where the hand is situated in front of the torso, the arm is relatively well configured to move in arbitrary directions (Figures 3A, B). Hence the isotropy of these ellipsoids is relatively high compared to when the hand is located outside of this region, as shown in Figures 3C, D.

Figure 3D depicts the upper limb in a singular pose. It is well known that the manipulability and the force ellipsoids will collapse into orthogonal line segments for manipulators. Similarly, for this arm configuration, the corresponding ellipsoid follows the same trend and elongates along the structure of the arm. In this pose, the force that can be sustained by the arm passes through the joints and subsequently generates no torques supporting the resulting ellipsoid.

### 4.2 Analysis of MFME and polytopes

**FIGURE 4:**
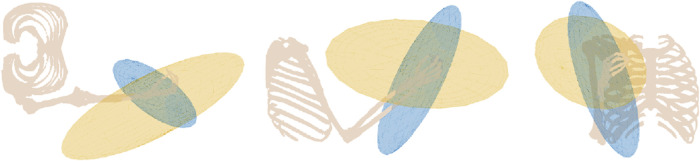
Calculated ellipsoids generated for the pose of the musculoskeletal model shown. The MFME is shown in blue, and the ellipsoid generated from the ray-shooting method polytope is shown in yellow.

From the vertices that form the polytope, the information contained can be simplified to understand the directions of how the limb is capable of arbitrarily moving. For Figure 4, an ellipsoid is generated using the vertices that form the polytope where a least-squares approach^1^ is used to ascertain the general directions that the limb is most or least capable.

For particular poses, it can be seen from Figure 4, that the axes of the MFME ellipsoid are visibly misaligned to the polytope-fitted ellipsoid. For the principal axis, this difference is noted to be 98°. Additionally, the magnitudes associated with each principal axis of the polytope-fitted ellipsoid indicate a better balance of strength in all directions. This measure is based upon a measure of eccentricity as described by Bayle et al. (2001), where the singular values of the ellipsoids, obtained via singular value decomposition, are utilized. For this measure, a value closer to 0 describes an ellipsoid that resembles a sphere, indicating a better balance of strength in all directions, while a value closer to 1 describes an ellipsoid that is elongated along the principal axes, indicating a direction that is better capable of generating force in than the other axes. From Figure 4, the MFME ellipsoid possesses a value of 0.98, whilst the polytope-fitted ellipsoid possesses a value of 0.97. Furthermore, when comparing the magnitude of the principal axis of these ellipsoids, the polytope-fitted ellipsoid possesses a radii value that is 52% the length of the MFME ellipsoid.

### 4.3 MFME vs. force ellipsoid

Results from the previous analysis raised questions about why there is such a difference in the methods. As a result, the MFME is compared to a force ellipsoid commonly computed for robotic arms. These methods are based solely on limb kinematics (Yoshikawa, 1985).

**FIGURE 5:**
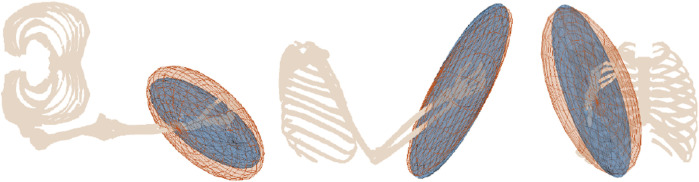
Calculated ellipsoids generated for the pose of the musculoskeletal model shown. The MFME is shown in blue and scaled force ellipsoid in orange.

Figure 5 compares the MFME and force ellipsoids. The results demonstrate that the MFME is heavily aligned with the force ellipsoid, with an angular difference of 16° for the principal axis, which suggests the MFME is primarily governed by limb kinematics.

### 4.4 Effect of muscular impairment

Assistive and rehabilitation robotics that meet the needs of users with physical impairment is an attractive use case for strength models. Here, strength models are compared to represent the change in whole-limb strength due to muscular impairment. For this analysis, the impaired muscle groups are the biceps, triceps and anterior deltoids. An impairment of each muscle group was simulated by limiting the activation to 10% of their full capability, and then a comparison has been made between the impaired and non-impaired strength profiles.

#### 4.4.1 Effect of muscle impairment on ellipsoid properties

**FIGURE 6:**
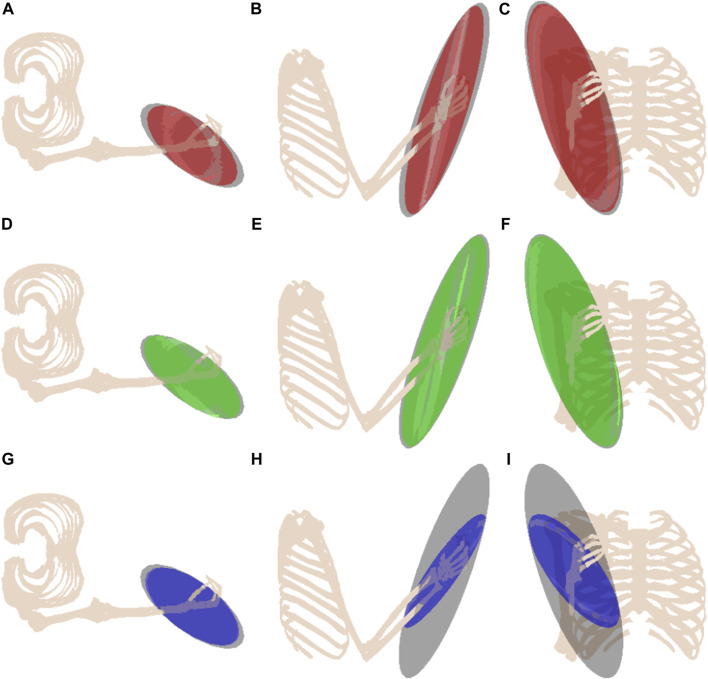
The effect of the muscular impairment on the muscular manipulability ellipsoid. The unimpaired ellipsoid is shown in grey, and the impaired muscle groups are shown as follows: biceps in red **(A–C)**; triceps in green **(D–F)**; deltoids in blue **(G–I)**.

Observing the MFMEs in Figure 6, it is interesting to note that the volume between the impaired bicep and tricep group is similar. In particular, this is illustrated in Figure 6A–F. Since muscles have the ability to “push” and “pull”, based on the activation unit-sphere constraint implies that −1 ≤ **a** ≤ 1, an impairment on the bicep can be compensated by the tricep and *vice versa*. The impairment of the deltoid is much greater than that of the bicep or tricep when comparing the difference in ellipsoid volumes, as highlighted in Figures 6H,I. This observation can be attributed to the amount of muscle force that can be generated by the deltoids’ antagonist muscle pair. From these illustrations, it is evident that the effect of this impairment is capable of altering the direction and magnitude of the ellipsoid’s principal axes, changing the direction in which the human is most and least capable.

#### 4.4.2 Effects of impairment on force output using polytopes

**FIGURE 7:**
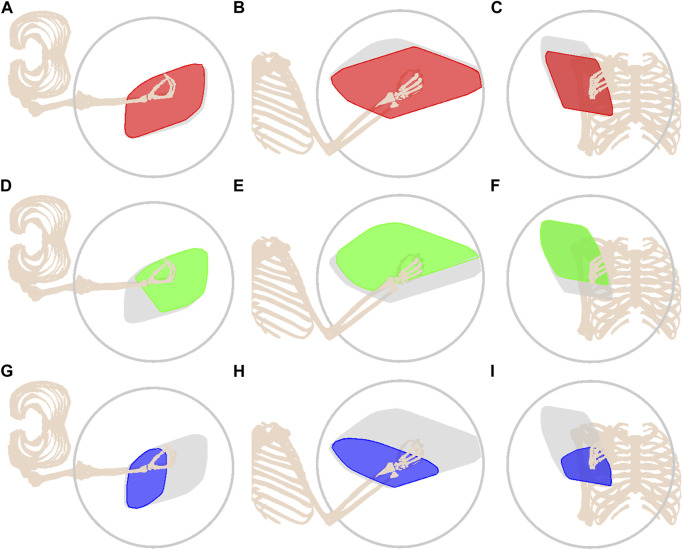
Effect of muscular impairment on the force polytope from different viewpoints. The unimpaired polytope is shown in dark grey, the upper force limit is shown in light grey where the grey circle corresponds to a radius of strength 300N, and the impaired muscle groups are shown as follows: biceps in red **(A–C)**; triceps in green **(D–F)**; deltoids in blue **(G–I)**.

Unlike ellipsoids, polytopes are able to capture the effect of removing one-half of an antagonistic muscle pair. For example, the bicep is responsible for generating a joint torque about the elbow, and its contraction would cause the hand to move towards the head of the model. Thus, theoretically, if the agonist bicep muscle is impaired, the force output about the elbow is reduced. This is supported by the resultant polytopes in Figure 7 comparing unimpaired and impaired profiles.

The impaired polytope profile better captures the result of impairment when compared to ellipsoid representations. Comparing Figures 6, 7, shrinkage is shown to occur on one side of the polytope, whereas the ellipsoid has minimal shrinkage since muscles can push and pull, allowing antagonist muscles to compensate for the impairment. The difference between ellipsoid and polytope representations is particularly striking when impairing the triceps. The polytope indicates a distinct loss of capability in the opposite direction of the bicep, supporting the agonist-antagonistic nature of the two muscle groups.

### 4.5 Consistency of polytope and ray-shooting approaches

**FIGURE 8:**
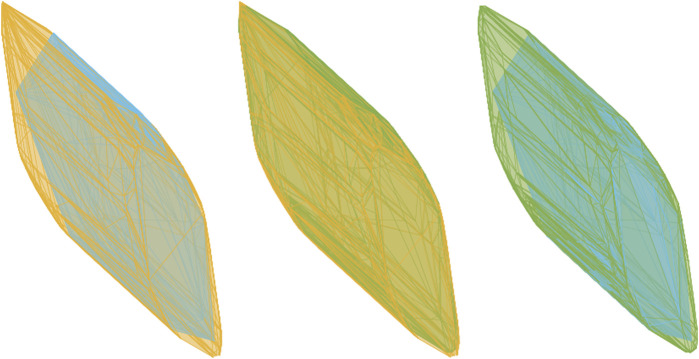
Three sets of two polytopes are generated using different methods, illustrating the discrepancies that exist. For all plots, the HPSM polytope is shown in yellow, ray-shooting method with the unit sphere discretized in cyan and a combination of the unit sphere discretized with the vectors representing the vertices of the HPSM polytope in green.

The HPSM polytope and ray-shooting methods are compared in terms of the consistency of the strength profiles they produce. First, the ray-shooting directions specified using an evenly distributed set of discrete directions are compared. The two polytopes, shown in Figure 8 on the left, show the HPSM polytope being visibly larger. The computation of the polytope volume shows a 12.5% difference.

Second, the comparison is repeated, this time by using the normalized vertices of the HPSM polytope as the directions for the ray-shooting method. The two polytopes, shown in Figure 8 in the centre, illustrate that by aligning the directions of the ray-shooting method with the vertices of the HPSM polytope, an improved consistency is achieved. In this case, a volume difference of 1.5% is calculated. This still represents a significant difference in the two methods.

The third comparison shown by the rightmost illustration in Figure 8 depicts two polytopes generated from the ray-shooting method. The difference between the two polytopes is a result of the input vectors for the ray-shooting method. It is evident that the choice of input vectors influences the resulting polytope. For the polytopes shown in the figure, a difference of 5.5% is recorded.

## 5 Discussion

The results show that there are clear differences in the methods used to calculate and represent whole-limb strength. For robotics applications utilizing musculoskeletal models, such as designing adaptive controllers for robotic rehabilitation, the choice of which representation is utilized has the potential to change the functional capability of the robot vastly. This is especially salient for robotic systems aiming to provide assistance corresponding to individuals’ loss of muscular capability.

Naturally, a difference in strength values calculated based upon (Carmichael and Liu, 2013) and the HPSM (Gouttefarde and Krut, 2010) will exist as a result of different formulations. The ray-shooting method presents the most accurate solution when calculating strength values since the equations of motion are not simplified. However, these strength calculations are limited to discrete directions, and when sampling a large sample space, like generating polytopes, the computational cost becomes intractable. While variations like HSPM can provide exact solutions for a complete strength representation, they struggle to enable real-time applications due to their computational complexity. Additionally, its current formulation makes assumptions regarding the equations of motion, which may skew the results. Alternative ray-shooting methods, which are adaptive based on a desired resolution or profile, may lower the computational time. However, achieving a high-accuracy polytope may reverse those computation time gains by increasing the total number of computations. Specifying a high precision for the proposed method in Skuric et al. (2022) results in a polytope that approaches the HPSM-generated ellipsoid. The discrepancy between methods could be a result of optimizing for the bias torques and subsequently optimizing for the muscle forces responsible for exerting the maximum output force whilst (Carmichael and Liu, 2013) attempts to find a muscle activation within a single optimization calculation.

Although the human upper limb is a highly redundant system, simulations of muscular dysfunction across three muscles, i.e., triceps (long, lateral and medial) indicate that muscle dysfunction can significantly affect the strength profile. It is particularly apparent when examining the strength polytope, where polytope shrinkage is asymmetrical and corresponds to the operation of agonist-antagonistic muscle pairs. In contrast, the ellipsoid representation appears to be relatively insensitive to modelled muscular impairment, demonstrated by minimal changes for both the ellipsoid volume and principal axes. This suggests that the evaluated ellipsoid strength profile is unsuitable for representing the change in whole-limb strength due to muscle dysfunction.

Strength results using the ellipsoid method were substantially different from the other methods compared. This is not unexpected, as significant assumptions and simplifications are required, limiting their applicability. Ellipsoid methods assume a unit-norm input, in our case, a unit-sphere of muscle activations. This is not a true neuromuscular constraint when performing physical tasks, and hence the strength profiles generated using the ellipsoid methods cannot be treated as a high-fidelity or perhaps even a realistic measure of whole-limb strength. In fact, this constraint implies that muscle activations can be negative, or in other words, that muscles can push to produce torque around the joint. This is obviously a substantial simplification of the mapping from muscle activation to joint torque. However, the simplicity and efficient computation of the ellipsoid method possesses benefits. The representation is particularly convenient for determining strength isotropy and volume. It also makes visualization easier. The ellipsoid method for calculating strength aligned closely with the force-ellipsoid method, which is commonly used when analyzing robotic manipulators. This suggests its suitability for use in applications dependent on kinematics considerations.

A mathematical approach for evaluating human strength is detailed in Section 2 and Section 3, and then applied in Section 4. Based on the findings in Section 4, methods that employ principles that closely align with human physiology and real-world physics demonstrate results with the highest accuracy, capable of discerning the effect of muscle dysfunction on a strength profile.

For applications that require a complete representation that can run online, the method proposed in Skuric et al. (2022) is ideal. These applications could involve assistance-as-needed control schemes dictated by maximum or minimum strength over the entire polytope. Alternatively, the assistance-as-needed system could enhance human strength uniformly in all directions based on the current pose that is exhibited by the human (Petrič et al., 2019). A complete representation of strength can aid rehabilitation practitioners in comprehending the impact of muscle dysfunction. The ability to identify such impairments would subsequently aid in guiding treatment (Baillargeon et al., 2022). Furthermore, an understanding of overall human strength in varying poses enables those working within ergonomics to make information driven design choices for workflows.

High fidelity representations have been used for biomechanics to study muscle redundancy (Kutch and Valero-Cuevas, 2011; Sohn et al., 2019) and responses to stimuli (McKay et al., 2007). In these works, highly accurate representations are convenient for simulating human and animal capability when compared with utilising cadavers. This enables rigorous studies that work towards understanding muscle coordination and quantifying muscle dysfunction sensitivity.

In the case of analyzing muscle dysfunction visually, it can be difficult to ascertain when viewing the entire polytope. It may be more useful to only view a slice of the polytope aligned with anatomical planes such as the frontal, sagittal or transverse. Although, it is possible to calculate an entire polytope to obtain a profile, producing a profile using the ray-shooting method may take less computational time. Similar to polytope representations of strength, these profiles enable practitioners to develop programs that target specific muscle groups, ensuring that other muscles are not overcompensating for the impaired muscle.

Through the quantification of muscle capability, assistance-as-needed control schemes or robotics rehabilitation devices can tailor the user experience by adapting the level of assistance based on a percentage of their estimated strength in a specified direction. The ray-shooting method would be suitable if the system only needs to determine the compensation required in a particular direction. In this approach, both impaired and fully capable muscle models can be utilized, enabling the optimization process to be called twice. The resulting difference can then be employed by the control scheme to determine an appropriate level of assistance.

For the ray-shooting method, an incomplete representation, caused by too few sampled vectors, results in accurate point estimates along those specific directions. For example, if the goal was to produce a force profile representing a human applying a force to a surface, the representation would reduce all other directions to 0. However, if a uniformly sampled sphere is used as the directions of interest but has too few vectors, then when fitting a convex hull to these points, the polytope inaccurately reflects endpoint strength along unsampled directions due to discontinuities produced by the sparse estimates. Likewise, the approach proposed by Skuric et al. (2022) yields a polytope accurate in selectively optimized directions while exhibiting underestimated values in unoptimized directions due to user-specified lower desired accuracy. These underestimations are either a value of 0 since the vector is not considered to be of interest or an inter-point surface estimate based on the polytope representation. In an AAN framework, these underestimations of the humans’ capabilities are likely to trigger additional user assistance. Although robotic assistance has been shown to benefit the human Skuric et al. (2022), it has been postulated that if an operation is perceived to be too easy or challenging for the human to accomplish, it is likely to result in a reduced performance. Subsequently, users must ensure that the areas of interest utilised, whether it may be for maximum or minimum strength, reflect the capability of the human.

Unlike the polytope and ray-shooting methods, ellipsoid methods struggle to capture muscular capability, and their overestimation of muscle properties do not accurately represent the magnitude or direction of muscle loss. These assumptions make this representation easy to visualize and convenient for determining the strength isotropy and volume. However, this makes ellipsoid representations an unsuitable candidate for tasks that require high-fidelity visualisations of strength. These representations can still be applied for assistance-as-needed control paradigms that are designed to provide assistance through an understanding of relative strength (e.g., when assistance provided is a ratio related to the major and minor axes corresponding to the maximum and minimum directions of strength, respectively).

Within biomechanics, ellipsoid models have been fitted to experimental data obtained for the purpose of quantifying the overall strength magnitude and isotropy (McKay et al., 2007; Baillargeon et al., 2022). In this respect, the representation possesses benefits based on its mathematical properties, similar to the MFME representation. Unlike fitting an ellipsoid to measured data, the MFME representation is heavily influenced by the kinematics of the limb rather than the effect of the muscles. Consequently, users should be mindful in its application. Since the representation is visually aligned with representations for limb kinematics, it lends itself well to understanding the natural stiffness of the arm and the directions it is capable of resisting forces leveraging the geometry of the limb (Ajoudani et al., 2017).

Further research is required to explore static and dynamic optimization for the upper limb and to analyze the implications of the parameter set, including factors like passive muscle forces and activation dynamics. As previously stated, these factors were omitted so as not to confound findings during the comparison. Additionally, this paper does not provide comparisons with data obtained from a user case study, however, the results presented provide similar trends for the effect of muscle impairment when compared to Carmichael and Liu (2013). In future, it would be of interest to investigate how individuals perceive different representations in a physical human-robot setup, utilizing an adaptive control scheme based on the outputs of the strength modelling method. This research direction would provide valuable insights into human preferences and needs while providing an informed resource for facilitating improved interactions between humans and robots.

## 6 Conclusion

Musculoskeletal modelling presents a promising avenue for developing robotic systems that understand humans’ physical needs. This paper outlines existing methods for calculating and representing strength using musculoskeletal models. Three prevalent methods for calculating and representing strength are compared: Point, Polytope, and Ellipsoid. These methods are compared to generate insight into human strength and how changes in the model affect the representations produced. It then details the differences and relative advantages of the existing methods, discussing the appropriateness of each method for particular applications.

The results demonstrated clear differences in the methods compared, indicating that careful consideration is required when choosing which methods to utilize in robotic applications. In particular, the ellipsoid method produced substantially different profiles than the other methods. This is attributed to the simplifications and biomechanical inconsistencies intrinsic in the ellipsoid representation. However, the ellipsoid method for calculating strength was closely aligned with the force-ellipsoid method, suggesting its suitability for use in applications is dependent on kinematics considerations.

Both the point representation (using the ray-shooting method) and polytope (using the HPSM method) showed alignment in their results and captured the effects of muscular impairment on whole limb strength. Small differences in strength results were observed, warranting additional research into the implications of both methods.

## Footnotes

1 Ellipsoid fit: https://www.mathworks.com/matlabcentral/fileexchange/24693-ellipsoid-fit
